# Editorial: Advanced oral disease therapy: approaches, biotechnology, and bioactive materials

**DOI:** 10.3389/fbioe.2023.1211443

**Published:** 2023-05-24

**Authors:** Hai Zhang, Xing Wang, Xianqi Li, Yuan Yin, Jianyun Zhang, Xiaoxuan Zhang

**Affiliations:** ^1^ Department of Restorative Dentistry, University of Washington, Seattle, WA, United States; ^2^ Shanxi Medical University School and Hospital of Stomatology, Taiyuan, China; ^3^ Department of Oral and Maxillofacial Surgery, Matsumoto Dental University, Shiojiri, Japan; ^4^ State Key Laboratory of Military Stomatology, Department of Periodontology, Fourth Military Medical University, Xi’an, China; ^5^ Department of Oral Pathology, Peking University School and Hospital of Stomatology, Beijing, China

**Keywords:** biomaterial, bioengineering, biomimetic, regeneration, biotechnology

In recent years, the advancement in biotechnology has enabled much improvement in quality and outcomes of medicine. These innovations have also drawn great attention in dental research fields. Regenerative medicine such as tissue (bone and tooth) engineering has been a hot topic in oral and craniofacial research for a few decades. Cells, signaling molecules and scaffold materials are three key components of tissue engineering approaches. Almost everyday new bioengineering approaches are proposed and tested for various diseases and treatment in dentistry.

Stem cells such as mesenchymal stem cells (MSCs) are multipotent and may differentiate into different cell types for tissue repair and regeneration. However technical issues including phenotype consistency, host immune response and potential tumorigenicity are still not completely resolved. Extracellular vesicles (EVs) originate from cellular endosomes and contain bioactive molecules to target cells by paracrine. It is known that these EVs are one of the major mediators of stem cells leading to their biological effects. MSC-derived EVs (MSC-EVs), instead of MSCs, may be potentially used in tissue repair and regeneration. The current status and future therapeutic applications of MCS-EVs in oral and craniofacial tissue regeneration are discussed in a review (Liu et al.). EVs secreted by human gingival MSCs (hGMSC-derived EVs) were shown to promote osteogenesis and neovascularization *in vitro* and *in vivo* (Wang et al.). The roles of stem cell-derived EVs and non-stem cell-derived EVs in bone tissue regeneration (critical-size defect model) are reviewed (Liu et al.). Engineering modified EVs may play important roles in future cell free EV-based bone tissue engineering therapies.

A new generation of scaffold materials has been developed for tissue engineering. A multifunctional structurally optimized hydrogel scaffold was designed by integrating polyvinyl alcohol, gelatin, and sodium alginate with aspirin and nano-hydroxyapatite (nHAP). The osteogenic of nHAP and anti-inflammatory function of aspirin were successfully synergized (Li et al.). Similarly, nanofibrous scaffold material can be modified to provide better coordinated regenerative endodontic treatment when pulp connective-tissue, dentin formation, revascularization and reinnervation need to be well orchestrated (Huang et al.). Silk fibroin nanoparticles were coated with genetically engineered cell membrane overexpressing toll-like receptor 4 and loaded with minocycline hydrochloride. These biomimetic nanoparticles demonstrated excellent targeted antibacterial and immunoregulatory effects *in vitro* and *in vivo* (ligature-induced periodontitis mouse model) (Deng et al.).

Other advancements in oral disease therapies are also made in recent years. Eldecalcitol, a novel active vitamin D3 analog, was shown to be effective on preventing alveolar bone loss in diabetes-associated periodontitis (Gao et al.). Targeted delivery of antitumor drugs has been recognized as a promising therapeutic modality to improve treatment efficacy, reduce toxic side effects and inhibit tumor recurrence. A controlled-release of an anti-tumor drug (apatinib) from supramolecular nanovalve-modified mesoporous silica was designed for targeted inhibition of osteosarcoma (Wang et al.). This *in vitro* study showed efficient release of antitumor drug and promising results for future osteosarcoma treatment. Enamel white spot lesions do not have effective yet conservative treatment methods. Synergistic remineralization of enamel white spot lesions was achieved by using mesoporous bioactive glasses loaded with amorphous calcium phosphate, which implies great potential for clinical application (Ren et al.).

The microbial composition and structural diversity of supragingival plaque on the surface of fixed prostheses were found to differ from that of normal natural crowns, with relatively high levels of periodontally related pathogens and higher microbial metabolism. The microflora on the surface of all-ceramic crowns was more similar to that of natural crowns than to that of porcelain-fused-to-metal crowns (Li et al.). Understanding the composition and differences between supragingival plaque biofilm microbes on the surface of fixed prostheses and natural crowns can provide patients with targeted guidance to focus on oral hygiene habits, reduce the risk of periodontal diseases and improve the success rate of fixed prostheses.

In addition to biotechnology, digital technology has also been applied in oral disease treatment, such as implant therapy. Additive manufacturing (AM) can enable the direct fabrication of customized physical objects with complex shapes, based on computer-aided designs. The applications of AM technologies in oral implantology, including implant surgery and restorative products, was reviewed (Huang et al.). Through the use of AM technology, personalized implant treatment for individual patients can be achieved.

With the quick advancement of therapeutic approaches, biotechnology, and bioactive materials, it will not be too long before precision and personalized dentistry become reality.

